# Digital health application integrating wearable data and behavioral patterns improves metabolic health

**DOI:** 10.1038/s41746-023-00956-y

**Published:** 2023-11-25

**Authors:** Ashkan Dehghani Zahedani, Tracey McLaughlin, Arvind Veluvali, Nima Aghaeepour, Amir Hosseinian, Saransh Agarwal, Jingyi Ruan, Shital Tripathi, Mark Woodward, Noosheen Hashemi, Michael Snyder

**Affiliations:** 1 January AI, Menlo Park, CA USA; 2 Stanford University, Stanford, CA USA

**Keywords:** Lifestyle modification, Patient education

## Abstract

The effectiveness of lifestyle interventions in reducing caloric intake and increasing physical activity for preventing Type 2 Diabetes (T2D) has been previously demonstrated. The use of modern technologies can potentially further improve the success of these interventions, promote metabolic health, and prevent T2D at scale. To test this concept, we built a remote program that uses continuous glucose monitoring (CGM) and wearables to make lifestyle recommendations that improve health. We enrolled 2,217 participants with varying degrees of glucose levels (normal range, and prediabetes and T2D ranges), using continuous glucose monitoring (CGM) over 28 days to capture glucose patterns. Participants logged food intake, physical activity, and body weight via a smartphone app that integrated wearables data and provided daily insights, including overlaying glucose patterns with activity and food intake, macronutrient breakdown, glycemic index (GI), glycemic load (GL), and activity measures. The app furthermore provided personalized recommendations based on users’ preferences, goals, and observed glycemic patterns. Users could interact with the app for an additional 2 months without CGM. Here we report significant improvements in hyperglycemia, glucose variability, and hypoglycemia, particularly in those who were not diabetic at baseline. Body weight decreased in all groups, especially those who were overweight or obese. Healthy eating habits improved significantly, with reduced daily caloric intake and carbohydrate-to-calorie ratio and increased intake of protein, fiber, and healthy fats relative to calories. These findings suggest that lifestyle recommendations, in addition to behavior logging and CGM data integration within a mobile app, can enhance the metabolic health of both nondiabetic and T2D individuals, leading to healthier lifestyle choices. This technology can be a valuable tool for T2D prevention and treatment.

## Introduction

37.3 million adults in the U.S. have diabetes; 95% of those have T2D^1^. Another 96 million U.S. adults have prediabetes; 5–10% of that population becomes diabetic every year^2^, and it is estimated that 70% of individuals with prediabetes will convert to T2D over their lifetime^3^. The annual cost of diabetes in the USA is $327b. Individuals with diagnosed diabetes incur $16,752 in health care expenses per year, which is 2.3 times greater than individuals without diabetes^4^.

Three major studies in the USA, Finland, and China have shown that lifestyle interventions, including dietary weight loss and physical activity, can prevent diabetes progression in high-risk individuals by 51–58% from 2.9 to 6 years after the original intervention^5–7^, with continued protection of 27–43% for up to 20 years^7–9^. Two additional prospective trials in India and Japan showed similar protection at 3–4 years post-intervention^10,11^.

Cornerstone features of the highly successful Diabetes Prevention Program include: individual case managers or “lifestyle coaches”; frequent contact with participants; a structured, 16-session core-curriculum that teaches behavioral self-management strategies, followed by additional classes and one-on-one meetings by phone or in person at least once per month; self-monitoring of weight, dietary intake, and physical activity; supervised physical activity sessions; tailoring of materials and strategies to address ethnic diversity; and an extensive network of training, feedback, and clinical support^12^. Due to its success, this approach has been widely adopted as the standard of care in individuals at risk for T2D. The interventions needed to implement this program, while cost effective, are resource-intensive and require contact with health care professionals/systems that may not be accessible to all, with reported participation rates as low as 2.6% due to lack of physician referrals and socioeconomic barriers^13^. During the first 4 years of implementation of the National DPP effort, only 39% of participants were retained at 44 weeks, and only 35.5% achieved the 5% weight loss goal^14^. Furthermore, while personalization is recommended, the current approaches offer no formal method by which to accomplish such personalization.

The explosion of new technologies that enable continuous glucose monitoring and activity tracking, as well as the widespread use of smartphones (currently used by 85% of US adults)^15^, provides a unique opportunity to leverage these technologies to significantly enhance the efficacy and practicality of lifestyle interventions. Technology-enabled diabetes self-management approaches have gained traction as supplements to traditional diabetes self-management models and implementation of DPP-based diabetes prevention approaches. Phone-based apps allow users to log fingerstick^16–18^ glucose values, body weight, food eaten, and physical activity, with some apps accessing large nutritional databases that allow patients to determine calories and carbohydrates consumed when the user enters food items or scans a barcode. Wearable devices log steps, minutes of activity, miles attained, and heart rate changes, as well as estimate calories burned^19^.

Continuous glucose monitors (CGM) are wearables that, via alarm features and real-time feedback to the user about glucose trends, were initially shown to lower both hyper and hypoglycemia in T1D and multidose-insulin-treated T2D^20–27^. Recently, significant but modest benefits (HbA1c −0.29% vs FSBG) were also shown in non-insulin-treated T2D^28^. There has been no formal evaluation of CGM as a tool for enhanced lifestyle modification as a method to treat or prevent T2D. Several small proof of concept studies have been published, showing that individuals with T2D using CGM chose lower glycemic index foods^29^, increased physical activity^30^, decreased caloric intake, lost weight, and demonstrated decreased postprandial glucose levels^31^. Among individuals with prediabetes, there is only one published study addressing the role of CGM in promoting behavior change, and, while it showed greater dietary self efficacy, neither weight nor glycemic measures were reported^32^.

The benefits of CGM as a behavior modification tool would be magnified if information relating food and activity choices were linked to glycemic responses and shared with the user, who could then learn from the combined data. Current technology, including wearable devices that simultaneously and continuously track multiple health metrics and mobile apps that can integrate data from wearable devices, have the potential to revolutionize behavioral approaches to diabetes treatment and prevention. While the current approaches to lifestyle management have proven successful, they are not tailored to individuals, who may respond differently to nutrients and activity. Indeed, several studies showed that glycemic responses between individuals differ following exposure to the same foods^33–35^, which may reflect a variety of interindividual biologic differences such as microbiome^35^, genetics^34^, and underlying physiology such as insulin resistance and beta cell function^35^. Adherence to diets and physical activity also varies between individuals due differences in personal preferences and environmental factors^36^.

Here, we sought to determine if a novel digital technology-based program, in which millions of CGM and other health data points were used to provide individualized feedback and tailored recommendations based on a user’s personal data patterns could improve lifestyle choices and metabolic health. We hypothesized that this approach, by inducing behavior modification that included healthier eating and physical activity, and took into account personalized glycemic responses and preferences, would promote weight loss, increase physical activity, and reduce hyperglycemia, which are important for treating and preventing T2D as endorsed by the American Diabetes Association and European Association for the Study of Diabetes for treatment of T2D^37^.

## Results

### Data collection and Cohort

**Fig. 1:**
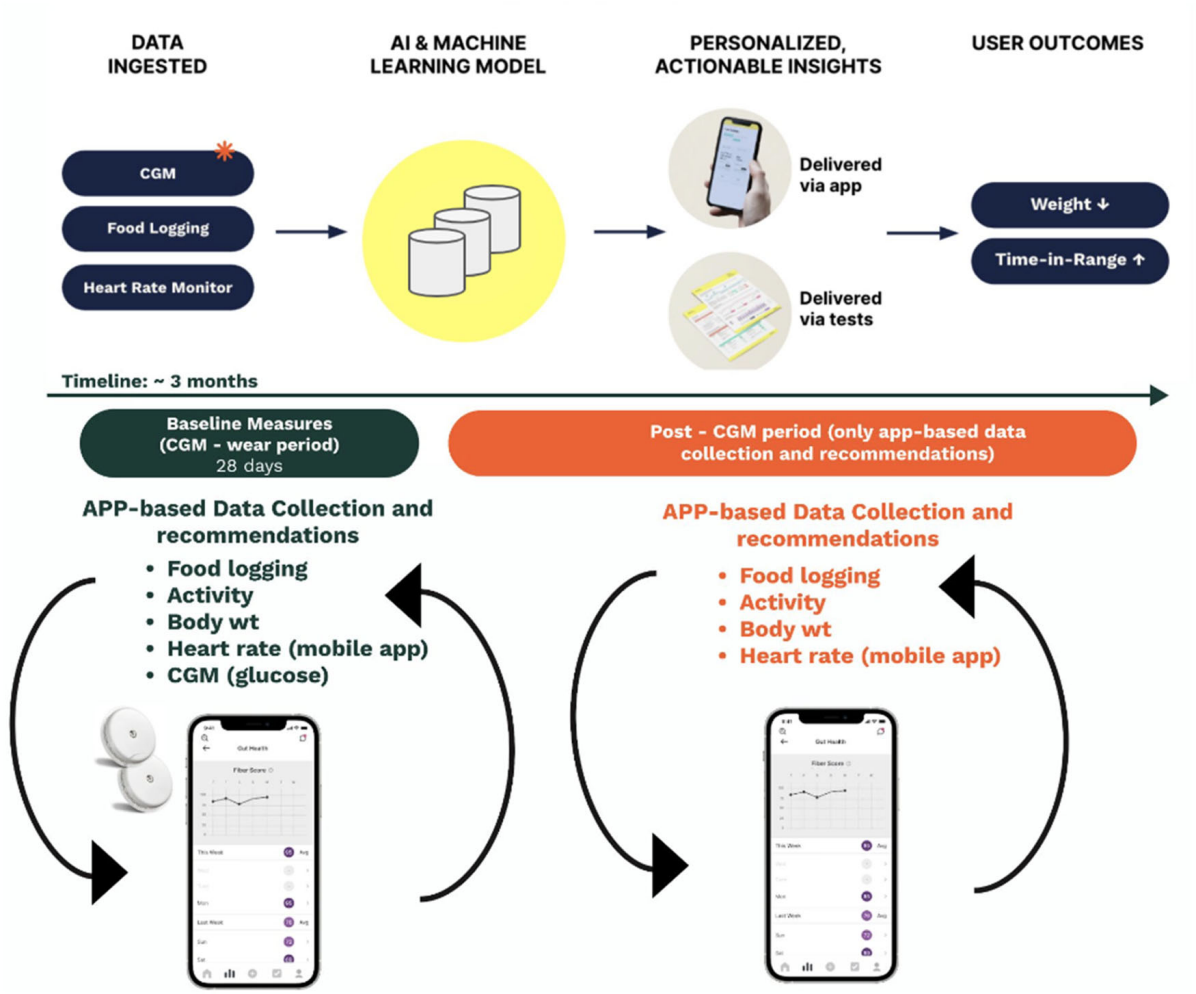
Season of Me Program Overview.

To assess whether the combination of wearable and machine learning data can be used to provide effective and personalized lifestyle recommendations, we collated a dataset from a cohort of *N* = 2217 individuals. These individuals were participants who enrolled in the Season of Me program, and agreed to provide retrospective de-identified data. Need for informed consent was waived (Advarra Internal Review Board). The Season of Me program was designed to leverage technologic advances to improve glucose time in range and weight loss in individuals with, or at risk for, T2D. Participants used a mobile application (“January AI app”) and wore a CGM (Freestyle Libre, Abbott) and HR monitor (Apple Watch or Fitbit) for 28 days (Fig. 1).

**Fig. 2:**
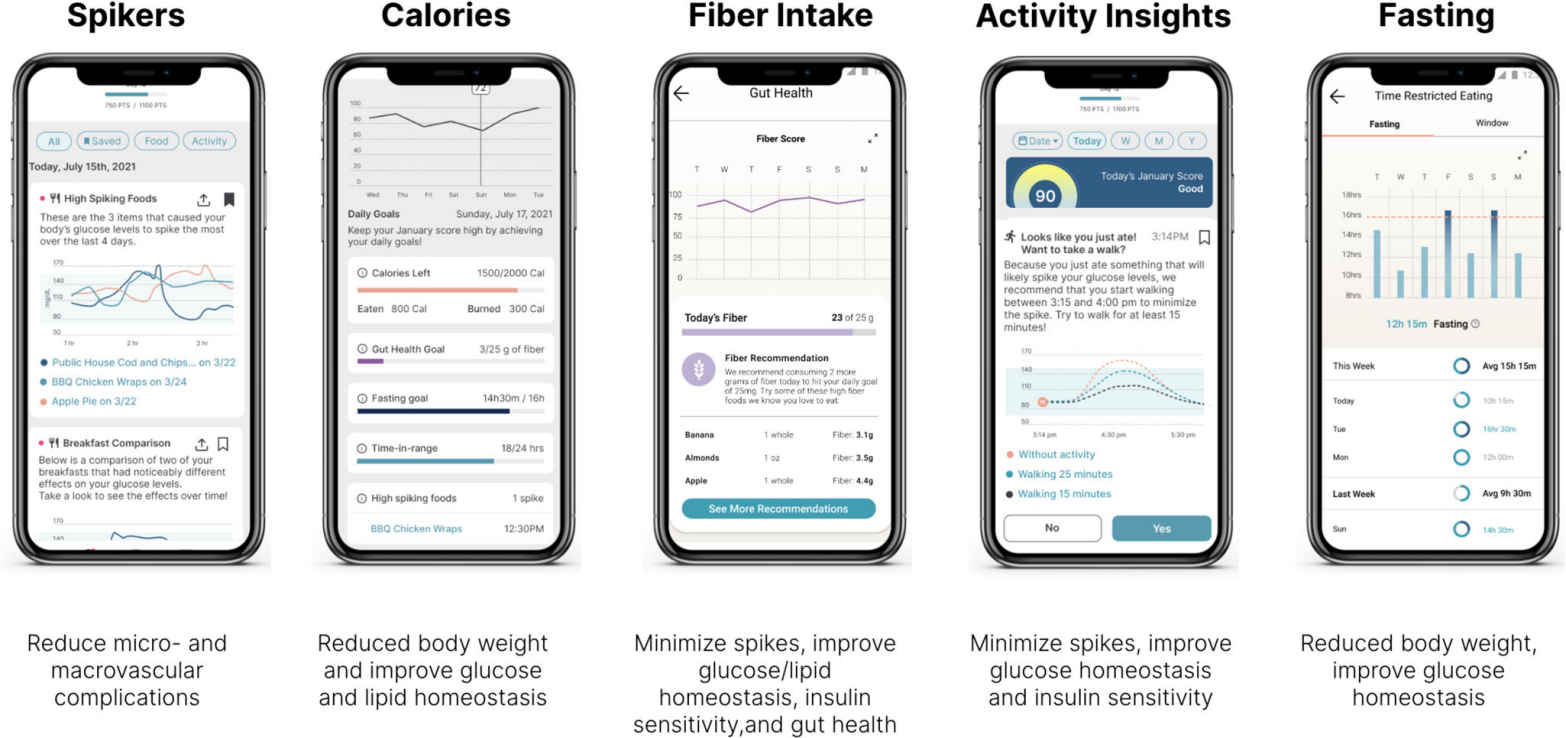
User experience with data synthesis, feedback, education, and personalized health recommendations based on previously recorded information.

The January AI app integrated CGM and HR data with user-entered diet and activity data. In addition to providing integrated response data back to users so that they would learn how lifestyle choices influenced their glucose patterns (Fig. 2), the app provided AI-based individualized recommendations (Figs. 1, 2, Methods).

Following the initial 4 weeks, an 8-week second phase followed, during which participants had the option to continue using the app without the use of CGM or HR monitor, relying only on personalized recommendations generated by the mobile app. Glucose analyses included 1066 participants who had a sufficient quantity of CGM data capture, consistent food logging, and regular body weight tracking.

Requirements for sufficient CGM data capture were: At least 70% CGM coverage on at least half of the days at the beginning (days 3–7) and the end (last 14 days) of the 28 day period. Requirements for consistent meal logging were: Active logging of all meals during the first 7 days, as well as the last 14 days. Requirements for regular body weight data tracking were: At least one body weight measurement in the first 7 days, and at least one body weight measurement in the last 14 days. Heart rate (HR) data was available throughout the entire period on all participants. Weight data was analyzed for 567 participants who met weight tracking criteria for inclusion (Methods). Over 27 million data points were captured across participant logs, HR, and CGM data. The majority of the participants were either normoglycemic (*n* = 746) or had prediabetes (*n* = 206), and a smaller subgroup had non-insulin-treated T2D (*n* = 94). The cohort was 49% male and 51% female, with an average age of 49 ± 11.5 years. Ethnicity data was not collected.

### Suboptimal control individuals showed notable TIR improvements, most significantly in healthy nondiabetics

Time in range (TIR) refers to time spent in the following glucose ranges according to previous recommendations: 70–180 mg/dL for those with T2D, and 70–140 mg/dL for those without T2D^24,25,38^. TIR was compared between the end of the 28-day program (defined as days 14–28), versus baseline (defined as days 2–7, excluding day 1 due to known inaccuracies in CGM readings during the first 24 hs of use).

The group as a whole (*n* = 1066) demonstrated relatively high baseline TIR, measuring 82% among T2D (70–180 mg/dL range) and 91% among those with prediabetes and healthy nondiabetics (70–140 mg/dL range), consistent with prior studies^24,25,39^. We believe that 70–140 mg/dL is more appropriate for individuals with normoglyemia or prediabetes since a target range should represent a range that is not already attained by nearly all individuals. Furthermore, since CGM is currently only approved for diabetes there has not yet been an official target range established for these groups.

**Fig. 3:**
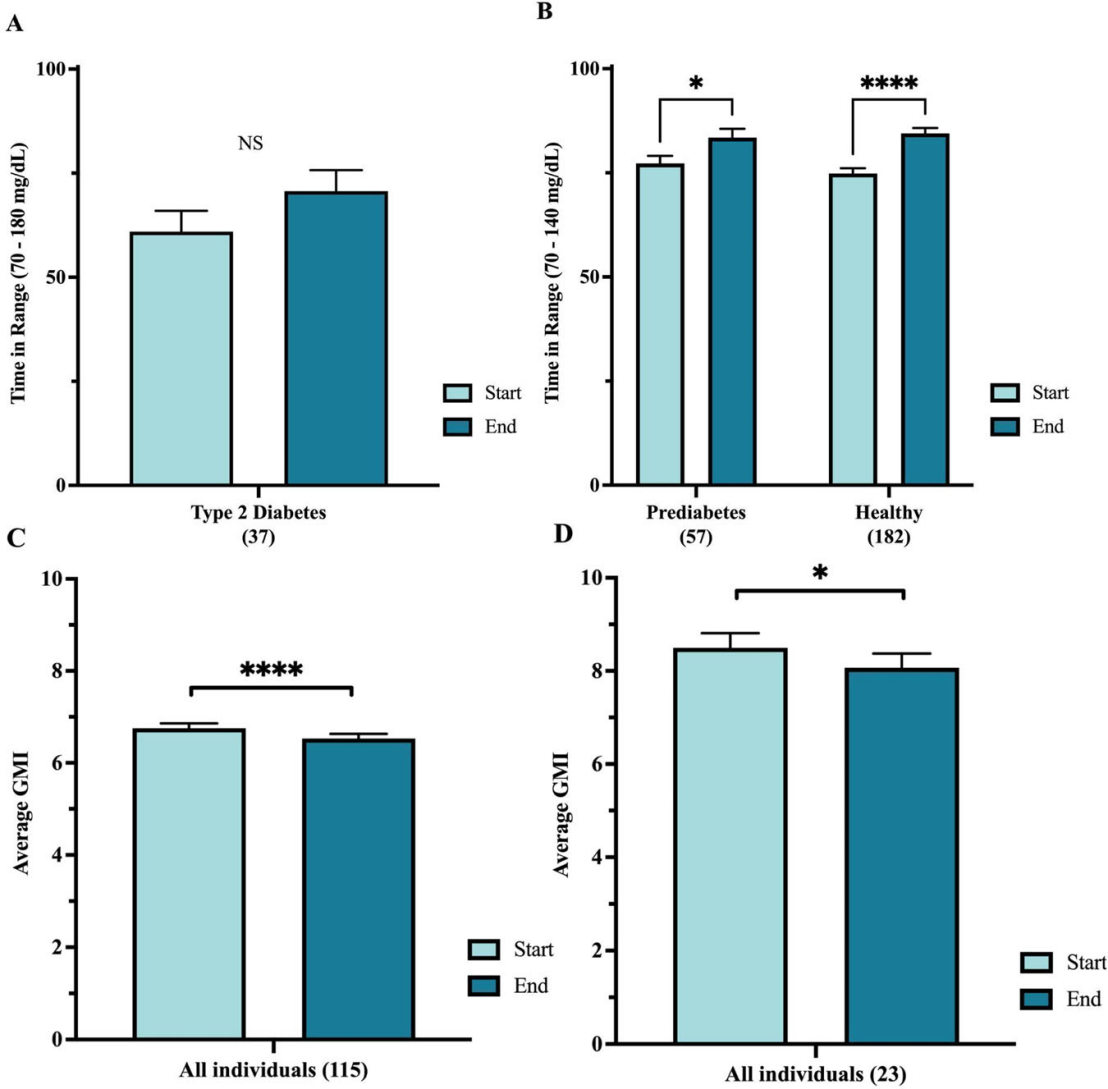
Change in level of glycemia in individuals with suboptimal control at baseline. **A, B** Time in range for individuals starting with <90% TIR defined as 70–180 mg/dL for T2D (A) and 70–140 mg/dL for prediabetes and healthy (**B**, **C**, **D**): GMI in individuals starting with GMI > 6% (**C**) and >7% (**D**).

TIR did not significantly improve for the group as a whole. However, individuals with suboptimal baseline control, defined as <90% TIR, showed notable improvements. When considering those with suboptimal control at baseline, defined as <90% TIR, those with T2D (*n* = 37) increased TIR by 9.8%, and individuals with prediabetes (*n* = 57) and healthy nondiabetics (*n* = 182) increased TIR by 6.2% and 9.6%, respectively (Fig. 3). For those with baseline TIR of <70%, improvement was even greater, ranging from 13.2% in T2D (*n* = 17), 9.6% in prediabetes (*n* = 9), and 22% in healthy nondiabetics (*n* = 51, *p* < 0.0001). Due to low numbers in these subgroups, only the healthy nondiabetic group reached statistical significance.

### Significant reductions in Glucose Management Indicator GMI, hyperglycemic events, and glycemic variability were observed across subgroups

Glucose Management Indicator (GMI) is a metric that uses CGM data to estimate HbA1c (ADD REFERENCE). Like TIR, GMI changes were evaluated in those with suboptimal values at baseline, defined as either >6% or >7%, irrespective of glycemic category. Significant GMI reductions were observed among participants with suboptimal values at baseline. Among those with baseline GMI > 7% (*n* = 23), GMI decreased by a mean of 0.43% (*p* < 0.001) and among those with baseline BMI > 6% (*n* = 115) GMI decreased by a mean of 0.22% (*p* < 0.00001). GMI reductions were similar and statistically significant in all glycemic subgroups (healthy nondiabetic, prediabetes, T2D) for both analyses (Fig. 3).

**Fig. 4:**
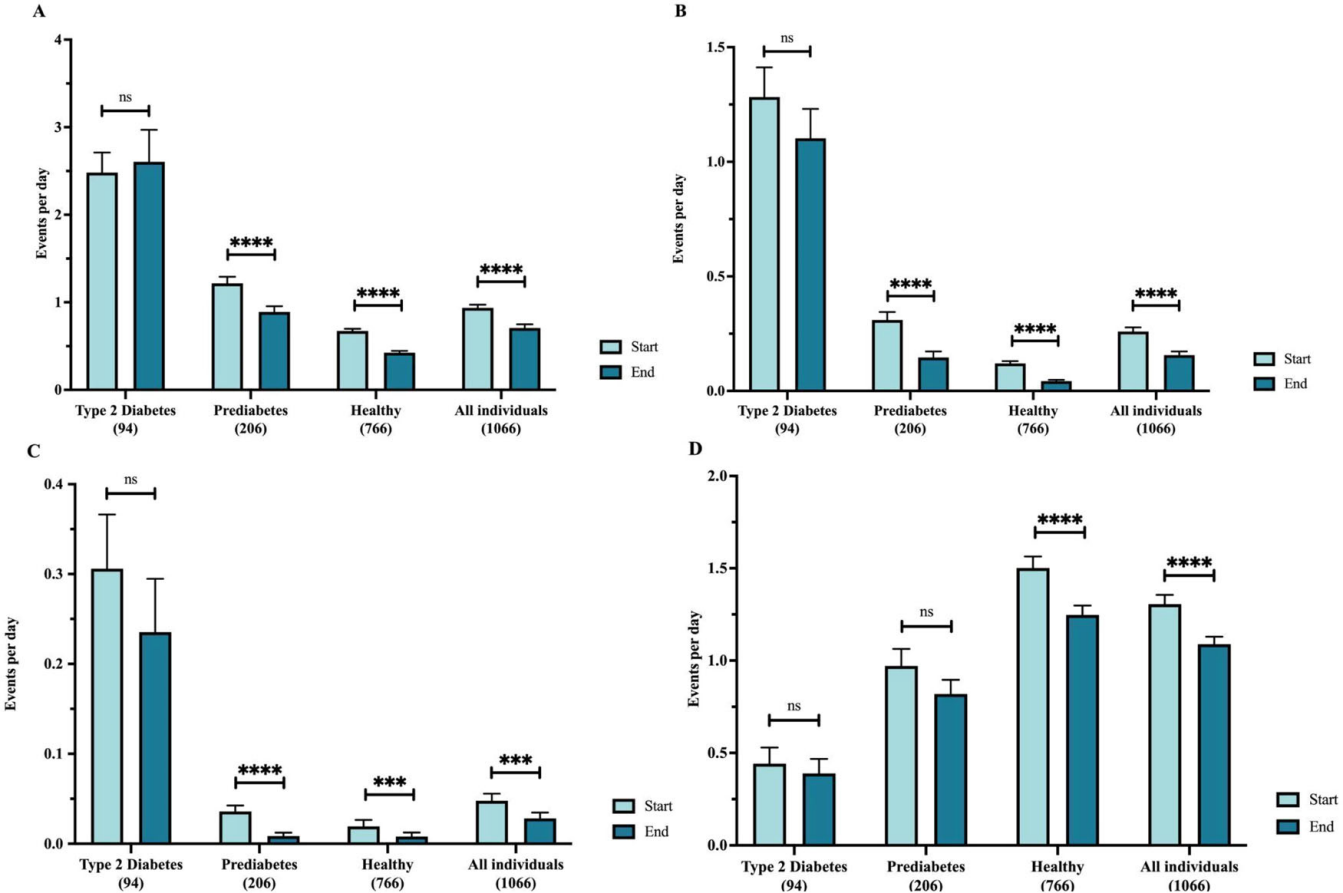
Change in hyperglycemic and hypoglycemic events/day. (**A**) Events > 140 mg/dL; (**B**) Events > 180 mg/dL; (**C**) Events > 250 mg/dL; (**D**) Events < 70 mg/dL.

**Fig. 5:**
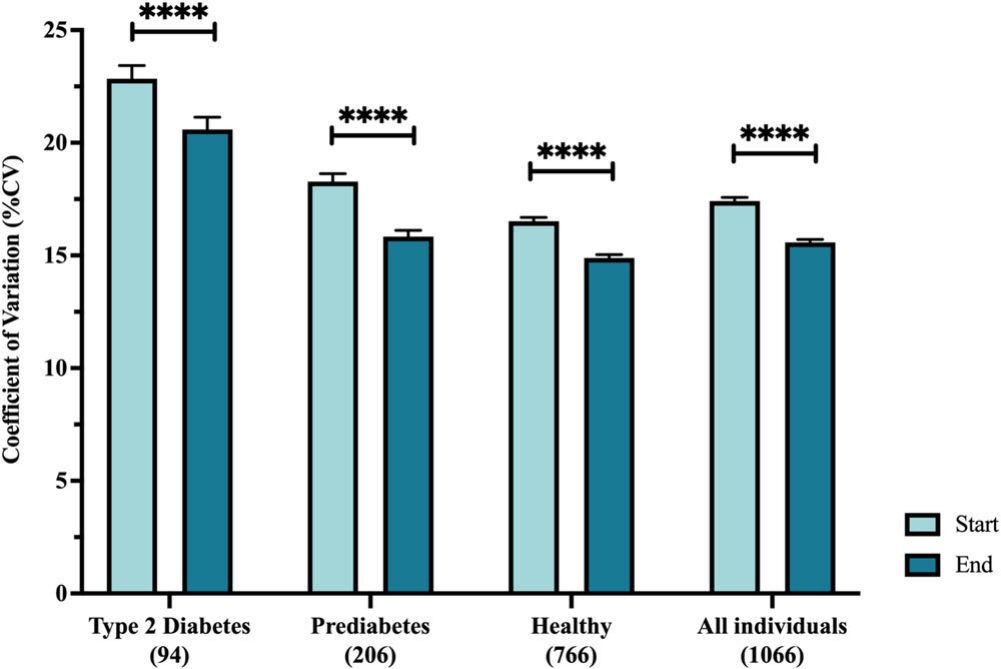
Change in glycemic variability measured as coefficient of variation.

In addition, the mean number of hyperglycemic events per day decreased in the cohort as a whole (*p* < 0.001): decreases in hyperglycemic events >250 mg/dL, >180 mg/dL, and > 140 mg/dL were 42%, 38%, and 24%, respectively, with the largest percent decreases in the healthy and prediabetes subgroups (Fig. 4). Decreases were present in all subgroups for all hyperglycemic events, with the exception of events > 140 which were not decreased in the T2D subgroup. Hypoglycemic events <70 mg/dL also decreased in the cohort as a whole, reaching statistical significance in the healthy nondiabetic subgroup (Fig. 4). Lastly, glycemic variability, measured as coefficient of variation, decreased significantly in all subgroups by an average of 13.5% (Fig. 5).

### Season of Me program aided weight loss, especially in higher starting weights and T2D participants

**Fig. 6:**
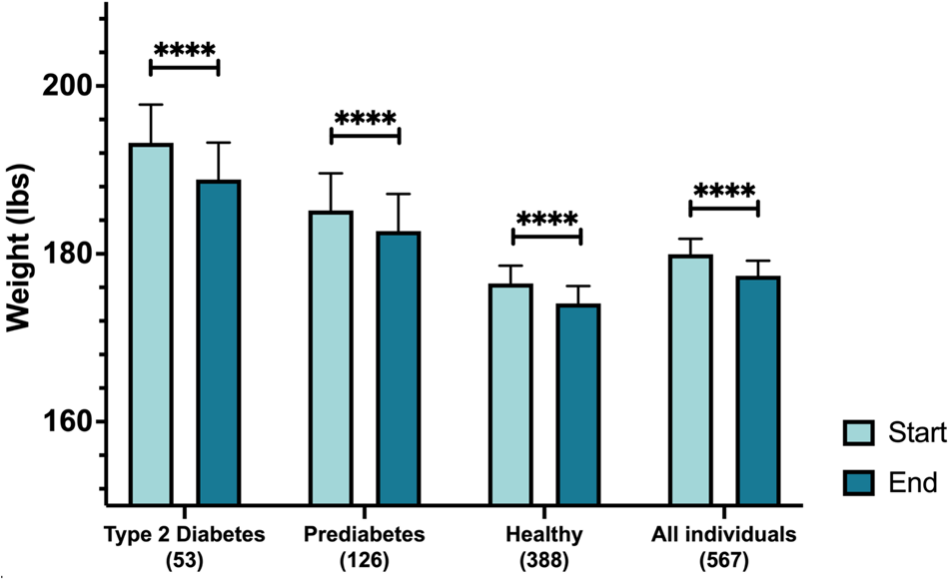
Weight loss at 4 weeks in those who logged at least one weight after baseline weight.

**Fig. 7:**
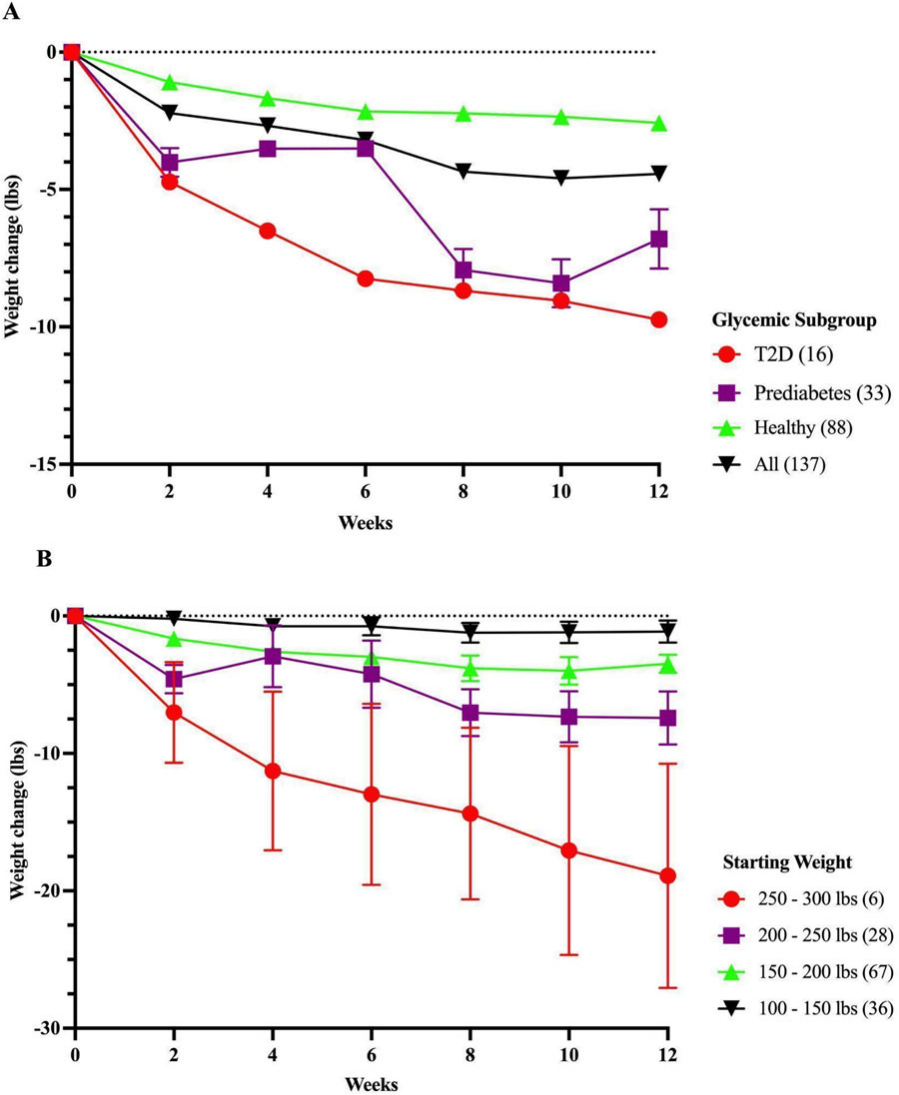
Weight loss during Season of Me Program. (**A**) Weight loss according to glycemic subgroup (**B**) Weight loss in those who continued to log weight over 12 weeks.

To determine whether Season of Me promoted weight loss, we compared the last body weight measurement during the final 2 weeks of the program with the first body weight measurement at the beginning of the program (*n* = 567). All groups of individuals within this cohort significantly decreased their body weight over 28 days (*p* < 0.0001, Fig. 6). Overall, 75.5% of the 567 participants lost weight over the first 28 days, with an average of 2.5 lbs among nondiabetic and prediabetic individuals, and 4.4 lbs among those with T2D. Those who continued the program for 12 weeks (*n* = 137) lost an average of 4.4 lbs, with 2.6 lbs lost in healthy nondiabetics (*p* < 0.0001), 6.8 lbs lost in those with prediabetes (*p* = 0.003), and 9.4 lbs lost in those with T2D (*p* = 0.0007) (Fig. 7). Individuals with higher starting body weights lost the most weight: those with baseline weight 250–300 lbs who continued logging weight through 3 months lost a mean of 11.3 and 18.9 lbs at 4 and 12 weeks, respectively, and individuals with starting body weight of 200–249 lbs lost a mean of 2.9 and 7.4 lbs at 4 and 12 weeks, respectively (Fig. 7, Supplemental Table S1). Expressed as % loss from initial body weight, at 12 weeks, the % loss was 1.5, 2.3, and 5.1% in healthy nondiabetic, prediabetic and T2D participants, respectively; and was 2.0, 3.2, and 6.8% in those weighing 150–199, 200–249, and >250 lbs at baseline.

### Participants, especially healthy and prediabetic subgroups, significantly increased daily physical activity during the study

Participants were instructed to log all meals and physical activity during the first 7 days, as well as the last 14 days. Actual logging use, defined as logging at least two activities of any type per day, was 81% during days 1–7, and 43% during days 14–28. Activity was recorded in minutes per event and added up to a daily total. To account for changes in logging frequency at the end versus beginning of the study, the total daily minutes of activity was adjusted for the number of times a participant interacted with the app. From the beginning to the end of the study, the adjusted minutes/day of physical activity increased from 49 to 97 min. By subgroups, the healthy and prediabetes participants approximately doubled their physical activity (50–109 min/day and 45–73 min/day, respectively), whereas those with T2D did not change their physical activity (45–46 min/day). There was no correlation between minutes/day of physical activity and change in TIR in the group as a whole or in any subgroups.

### Heart rate as an objective measure of physical activity

HR data capture was 100% throughout the study. To objectively assess changes in physical activity, HR > 110 bpm quantified as min/day was assessed in the group as a whole (*n* = 1066) and among glycemic subgroups (healthy, prediabetes, T2D). Five individuals were excluded due to resting HR > 110 bpm which precluded using this measure to indicate physical activity. In the group as a whole, HR > 110 bpm increased from 28.8 to 32.2 min/day (*p* = 0.008). Among healthy normoglycemic individuals (*n* = 764) the increase was 2.9 min/day (*p* = 0.009), whereas in those with prediabetes (*n* = 204) it was 6.3 min/day (*p* = 0.02), and in those with T2D it was 0.40 min/day (*p* = 0.88). Detailed data are available in Supplemental Table S2.

### Participants demonstrated decreased caloric, carbohydrate, and sugar intake, and increased protein and fiber consumption

A key feature of the program was personalized food insights and recommendations based on integrating glycemic responses captured by CGM and food logged on the mobile app by the user. 526 of the 1066 participants logged foods during the specified time periods (the first 7 days for baseline, and the last 14 days for end-of-study data) and were included in the analysis. Although over 2200 participants began the program, many did not generate sufficient data to perform analysis, for example, non logging of food, HR, etc; these participants were excluded from analysis, leaving 1066 participants upon whom analysis was performed. To account for potential decrease in logging adherence, only days in which food was logged at least twice, and totaled at least 1600 calories, were included. Average daily calories and macronutrient intake (including sugar, fiber, and saturated fat subcategories) were calculated. Macronutrients were expressed as absolute values (grams), and as a percent of total calories. Participants across all subgroups demonstrated decreases in caloric intake, carbohydrate, sugar, and saturated fat intake, and increases in protein, total fat, and fiber intake (Table 1 and Supplement Table S3). Percent change in the relative intake from each macronutrient was decreased for carbohydrates (-2.6%), sugar (-12.3%), and saturated fat (-0.5%); and increased for protein (+3.45), total fat (+1.1%) and fiber (+7.9%), indicative of a dietary improvement.

**Table 1:** Percent change in nutrient intake from beginning to end of the Season of Me program.

	kcal	Carb: Total kcal	Sugar: Total kcal	Protein: Total kcal	Fat/ Total kcal	Sat. Fat: Total kcal	Fiber: Total kcal
**All**	-21.8	-2.6	-12.3	3.5	1.1	-0.5	7.9
**Healthy**	-22.9	-2.2	-13.1	5.4	1.6	-0.7	10.0
**Pre**	-21.0	-4.7	-11.2	-4.0	2.8	-2.8	7.9
**T2D**	-11.9	-4.8	-7.3	1.0	-3.5	-3.9	0.9

To account for potential bias due to decreased frequency of logging (min of two meals and 1600 kcal per day was required for inclusion), macronutrients are expressed as a proportion of total calories to demonstrate the relative intake of macronutrients which should be free of logging bias. absolute caloric and macronutrient intake is shown in supplemental data. Carbohydrate, sugar, and protein grams were multiplied by 4 to calculate calories, and fat and saturated fat grams were multiplied by 9 to calculate calories consumed for specific macronutrients. Fiber was expressed as grams:total calories.

Macronutrients expressed as macronutrient calories:total calories and fiber expressed as grams:total calories

## Discussion

This real-world study demonstrates that use of digital technology in combination with CGM can facilitate lifestyle interventions that yield improvement in glycemic measures, in the largest cohort to date.

Three studies have previously examined the use of CGM for lifestyle change T2D^29–31^ Cox et al.^29^ included only four patients and did not have a control group. Allen et al.^30^ utilized solely physical activity intervention. Yoo et al.^31^ randomized patients with poorly controlled T2D (baseline HbA1c of 9%) who were treated with insulin (60%) or noninsulin therapies (40%), to real-time CGM vs. self monitoring blood glucose (SMBG) and demonstrated significantly greater reduction in HbA1c (0.50%, *p* = 0.004) at 12 weeks in the CGM group, along with reduction in total daily calorie intake, greater weight loss, and increased physical activity. Wada et al.^28^ examined individuals with non-insulin-treated T2D with baseline HbA1c of 7.8%; the researchers did not measure lifestyle changes, but showed that use of flash CGM as compared to SMBG resulted in a reduction in HbA1c at 24 weeks (0.29%, *p* = 0.02).

Our results extend the prior studies by demonstrating that use of CGM with a digital app designed to enhance healthy lifestyle behaviors by informing users of the impact of food and activity choices on glycemic responses yields both behavior changes and glycemic improvement in individuals with prediabetes and even earlier stages of dysglycemia detected by CGM. Combination of CGM data with heart rate and activity information, as well as content and personalized insights, is a novel approach to management of metabolism, and yielded noteworthy results.

Glycemic improvement was observed in the group as a whole, as was reduction in glycemic variability, but the greatest improvement was evident in those whose glucose was not within the optimal range at baseline, defined as 70–140 mg/dL for those without diabetes and 70–180 mg/dL for those with T2D. This is the first study to evaluate personalization of lifestyle recommendations based on previously observed glucose patterns from CGM. The use of digital technology for diabetes treatment and prevention has been previously studied, with applications ranging from remote coaching to dissemination of educational programs such as the DPP^40^ Importantly, none of these studies have evaluated integration of CGM with lifestyle measures or coaching, especially in addition to presentations to subjects of the projected glucose impacts of specific foods/meals and/or activity patterns.

Like prior digital health apps, the one used in this study served as a virtual coach, offering recommendations and reminders that addressed many components of medical nutrition therapy as recommended by the American Diabetes Association^41^ These include choosing healthy foods, and tailoring recommendations to consider individual preferences and environmental constraints (access, budget, living situation). Most importantly, however, this technology system (CGM and mobile app) uniquely integrated glycemic excursions obtained from CGM with actual food consumed, which allowed for personalized rather than generic coaching. Consultation with a RD in order to develop an individualized eating plan is associated with HbA1c reductions of 0.3-2.0%^42^ In the present study, which spanned only 28 days and did not include any live coaching, GMI decreased by 0.43% in those with baseline GMI > 7%, and by 0.22% in those with baseline GMI 6–6.9%. In addition, hyperglycemic events (glucose spikes), glucose variability, and TIR improved in all subgroups ranging from healthy nondiabetic to T2D, although the small T2D subgroup reached statistical significance only for glycemic variability. Hypoglycemic events <70 mg/dL also decreased in the group as a whole, although, in subgroup analyses, statistical significance was reached only in the healthy nondiabetic subgroup.

Based on data collected, the observed glycemic improvements likely resulted from three lifestyle changes: (1) healthier food choices; (2) weight loss; (3) increased physical activity. While only 526 of the 1066 participants logged sufficiently to be included in the detailed dietary analysis, food log reviews showed a decrease not only in total calories, carbohydrates, sugar, and saturated fat, but also in the proportion of calories from carbohydrates, sugar, and saturated fat, with an increase in the proportion of calories from protein, healthy fats (fats other than saturated include mono and polyunsaturated), and fiber. Although we did not quantify changes in the intake of meals that led to glucose elevation, the observation that there were fewer hyperglycemic events and that TIR improved strongly suggests that glucose-elevating meals were decreased.

Weight loss is a cornerstone of diabetes treatment and prevention. In the present study, use of the CGM and mobile app promoted weight loss in >75% of participants who continued to log weight. The amount of weight lost was greater in those with T2D and those with higher baseline starting weight, who at 12 weeks lost up to 10.4 and 18.9 lbs, respectively. At 12 weeks, the % loss from initial body weight was 1.5, 2.3, and 5.1% in healthy nondiabetic, prediabetic and T2D participants, respectively; and was 2.0, 3.2, and 6.8% in those weighing 150–199, 200–249, and >250 lbs at baseline. Because not all participants logged weight throughout the study, this may selectively represent those who were more successful in losing weight, who owned a personal scale, or who were more motivated in general. Thus, the weight loss benefits in those who did not continue to log weight is not known. Nonetheless, it appears very likely that use of the CGM with app and self-weighing at least once after baseline is associated with progressive weight loss for at least 12 weeks of use. Longer term studies will be needed to determine whether this is sustained.

Physical activity also lowers glucose levels, and the participants in the SOM program demonstrated increased physical activity as measured both by self report (logging) and by an objective measure (HR > 110 bpm). Multiple previous studies have demonstrated that use of accelerometer or heart rate monitor increases physical activity^43,44^ Thus, it is not discernible whether simply wearing the HR tracker/accelerometer or receiving personalized activity recommendations from the app contributed to the observed increase in physical activity. Further, the nature and intensity of the activity, as well as nuances in HR change that more specifically address intensity and conditioning, were not available for this analysis. Ultimately, the observation that the technology-based intervention as described leads to behavior modification and clinical benefits is important. It should be noted that participants in both the healthy and pre-diabetes cohorts already had relatively high levels of baseline activity, which could have skewed the data *vis a vis* their inclination toward performing additional exercise; nevertheless, significant increases in physical activity among both groups was observed.

It is worth noting the significance of the glycemic improvements observed in the healthy nondiabetic group. That the observation group exhibited glycemic excursions that could be improved by diet might come as a surprise to many. CGM data on nondiabetics is scant in the literature. While the TIR is high in this group at baseline, in the current analysis, which is the largest cohort of nondiabetic individuals with published data on CGM, glycemia still improved in multiple metrics. Whether improved glycemia in this population prevents diabetes or improved health outcomes is not currently known, as diabetes prevention studies have been conducted in high risk individuals with prediabetes. The data presented in fact suggests that with the advent of CGM an even earlier stage of dysglycemic is detectable and can improve with lifestyle interventions. This is important as future studies should examine the long term health risks (T2D, cardiovascular disease) as well as the impact of diabetes prevention strategies in this group of individuals. Such studies, which will take years to conduct, will determine whether the improvements in glycemic profile would be beneficial to health and potentially reduce the risk for T2D.

The current findings have several limitations inherent in real world studies. First, there was no control group—data presented are based on the change from baseline to end-of-program and thus could represent a “placebo” effect from simply being enrolled in a program. A future randomized trial with a comparator group would extend and confirm the present findings. Second, the requirement to use a CGM means that it is likely that the participants in this study demonstrated high levels of self-efficacy, and thus it is not clear that results would translate into less self-motivated individuals who were recommended by a health care provider to engage in a similar intervention. Third, some of the data captured depends on adherence to logging. It is possible that participants did not log all food or activity; thus, alternate metrics were used, such as the ratio of macronutrient to total calories and HR > 110 min, which generally supported the logged metrics. Logging weight is also subject to success bias and thus may only be interpreted to reflect the weight loss of those who weighed themselves at least once after starting the program. Fourth, ethnicity was not collected in this cohort. It is important to recommend ethnically and culturally appropriate lifestyle interventions, particularly with regard to dietary recommendations, and thus improvement of this application would include capture of ethnicity and ethnic/culturally-sensitive recommendations if desired by the user. Fifth, the duration of intervention was 28 days. Longer duration studies will be required to ascertain the durability of behavior changes and glycemic benefits. Finally, weight reporting in this study was not comprehensive as weight loss was not a specific goal of our study and therefore weight monitoring was optional. Weight logging was not required, nor were users reminded to log or enter their weight. It is not appropriate to compare our study to the DPP in terms of weight reporting, as our study’s focus was on logging food and activity and measuring glucose levels with CGM.

In our participants without a diagnosis of T2D, we used thresholds for Time in Range (TIR) that were previously reported in the literature (70–140 mg/dL) or justifiable based on translation to Hemoglobin A1c (Glycemic Management Indicator (GMI)). Currently, there are no TIR thresholds for patients without diabetes, but we believe that they will be established in the future^38^ It is not clear whether glucose levels outside a prespecified range (eg 70–140 mg/dL) or above a given threshold (>180 mg/dL) in individuals who do not meet traditional criteria for diabetes lead to clinical consequences such as microvascular disease. It is also not clear whether glucose levels on CGM predict increased conversion rates to T2D in those who do not meet traditional OGTT criteria for prediabetes. Long-term studies will be required to ascertain these important questions. However, both mild glycemic excursions into prediabetic and diabetic range, and glycemic variability, which has been associated with increased risk for cardiovascular disease^45^ are more readily ascertained with CGM than with older methods of glucose measurement, and it is likely that future studies will reveal answers to the questions posed above regarding the prediction of clinical events according to specific CGM metrics in both T2D and earlier stages of dysglycemia.

In summary, we demonstrate that an app-based platform that integrates food and physical activity logging with CGM and HR data, and provides personalized lifestyle recommendations based on user input enhances healthy lifestyle practices and improves metabolic health in individuals with and without T2D. This nee technology improves glucose profiles even in individuals who by current standards are normoglyemic, and promotes weight loss in overweight and obese individuals, highlighting its potential for early intervention. Importantly, the ability to offer intervention without human coaching (with or without AI-enabled personalization) enables affordable scaling to large numbers of people, including those who are underserved or living in remote areas––thus helping to reduce the continually increasing prevalence of prediabetes and diabetes. In this respect, it will be important to optimize user engagement, including customization to varied ethnicities and socioeconomic levels, and to consider the needs of different age groups, including the elderly who often have more difficulty accessing in-person health care services, and to youth and young adults, who tend to be less adherent to traditional models of diabetes prevention and may selectively benefit from technology-based models. Overall, this approach and similar technology-based approaches have the potential to improve metabolic health at early stages, and may increase the efficacy of current practices to prevent and treat T2D through lifestyle modification.

## Methods

The methods were performed in accordance with relevant guidelines and regulations and approved by the Advarra review board.

### Participants

Individuals over the age of 18 years were eligible to participate. Those with a prior diagnosis of diabetes who were not taking insulin were eligible, as were those with prediabetes or no history of glucose abnormalities. There were no body weight or BMI restrictions. Only participants who signed a disclaimer to use deidentified data (DID) were included. An external review board (Advarra) confirmed that analysis of DID was exempt from requiring formal informed consent.

### The season of Me program for personalized metabolic health management

The Season of Me (SoM) program was designed to leverage technologic advances to improve glucose time in range and weight loss in individuals with or at risk for T2D. Participants paid to use a mobile-app (January AI) and wear a CGM (Freestyle Libre, Abbott) and HR monitor (Apple Watch or Fitbit) for 28 days (Fig. 1). The mobile app integrated CGM and HR data with user-entered diet and activity data, along with wearable-tracked HR. In addition to providing integrated data back to users, the program provided individualized recommendations based on data both logged by users and pulled from users’ wearable devices.

The first 14 of 28 days consisted of an experimental phase, during which participants were monitored using a CGM and a HR monitor. The initial 4 days of this period served as a baseline, during which participants continued their regular diet and activities. On Day 3, participants undertook a glucose shot test. Commencing from Day 5, time-restricted feeding was introduced based on a schedule suggested by the app, and specific food experiments were initiated (e.g., ‘Low Glycemic Load first meal’ on Day 6).

Upon completion of the 14-day experimental phase, participants received a personalized report. This report compiled health insights derived from the data collected during the experimental phase, and provided tailored recommendations aimed at improving glycemic control, focusing on the enhancement of TIR.

**Fig. 8:**
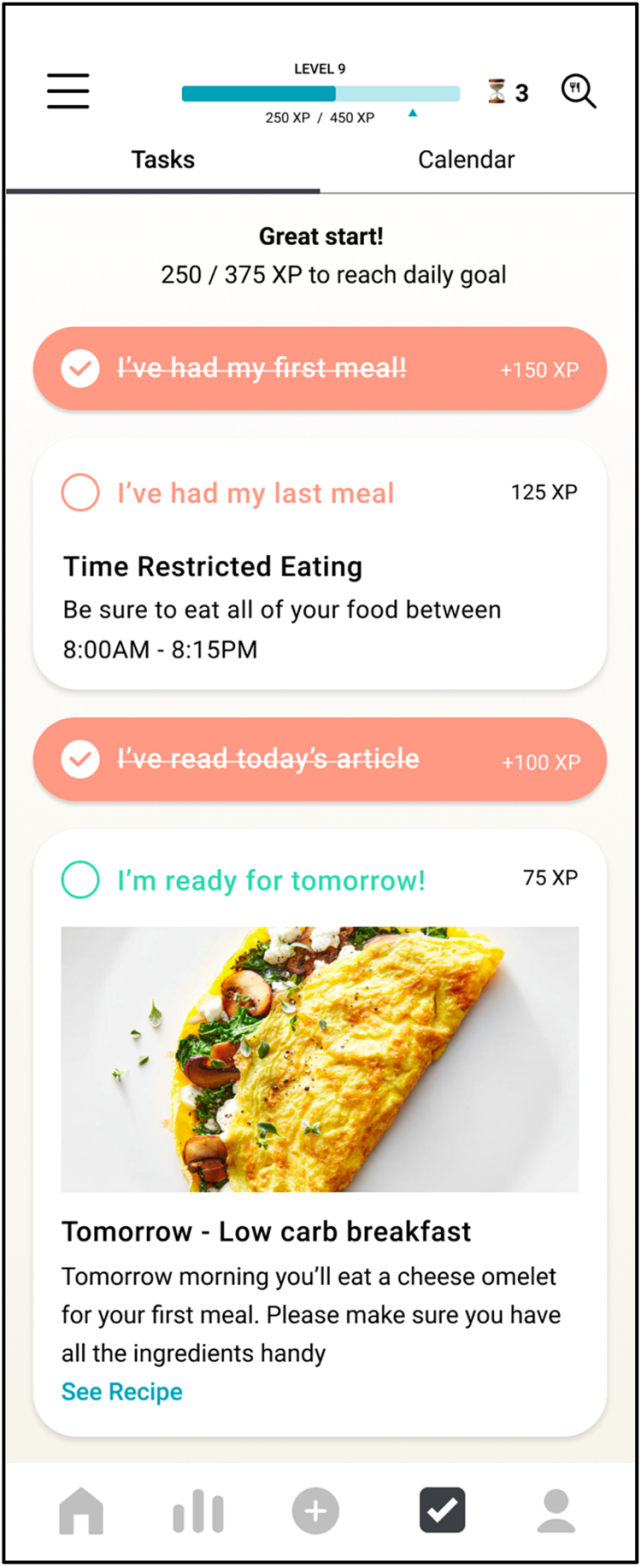
Example task card. Such a task card would have been presented to participants in the Season of Me program via their mobile application. Users can indicate whether or not the task was performed by toggling the button indicated.

Subsequent to the experimental phase, participants transitioned into a 15-day Time in Range Improvement Phase. During this period, daily tasks, structured as task cards within the mobile application, were assigned to participants. Each day involved the completion of 3–5 specific tasks, including mandatory reading of educational content (Fig. 8).

For Days 1–14, tasks predominantly consist of adherence assignments (for example, “Wear your HR monitor for at least 23 h today”); food experimentation tasks; and time-restricted eating tasks. From Days 15–30, tasks were primarily centered on interventions to enhance TIR. These encompass strategies such as carbohydrate reduction, caloric restriction, exercise regimens, time-restricted eating protocols, and mindfulness practices.

To facilitate participant learning throughout the 30-day journey, Insight Cards were provided via the app. These cards were categorized into Program-related Insight Cards and General Insight Cards. Program-related Insight Cards provided comparative analyses between different days and conditions (e.g., “Day 3 vs Day 4 vs Day 6”) to visualize the impacts of various interventions on blood glucose levels. General Insight Cards include Food Cards and Activity Cards, which illustrated the glucose and HR responses to a specific food item or physical activity, respectively.

Following the initial 28 day phase, a 2-month optional second phase followed, during which participants implemented the learnings without the use of CGM or HR monitor, relying only on personalized recommendations generated by the mobile app. These recommendations centered around the following five levers (Fig. 2):

1. Reducing spiking foods. Based on users’ food logs and CGM data, the app identified the foods that caused the largest increase in blood glucose, and offered alternative, lower-glycemic foods.

2. Calorie restriction. The app gave users personalized total caloric recommendations based on age, weight, height, sex, and physical activity at baseline, with recommendations to stay below the caloric requirement for weight maintenance.

3. Increasing fiber intake. After an observational period during which users’ baseline fiber intake was observed, the app suggested fiber-rich foods to increase daily fiber consumption to 21–25 and 30–35 g/day for females and males, respectively. The app not only identified users’ existing sources of fiber, but also suggested alternative foods with higher fiber content.

4. Increasing activity. After an observational period during which users’ baseline physical activity level was observed, the app suggested activity, especially post-meal activity, with the goal of reducing postprandial glucose spikes.

5. Increasing fasting period. The app recommended a 16-h fasting target to all users, tracking users’ fasting periods and comparing observed fasting activity against goal.

### Delivery of personalized food and activity recommendations

Health goals were addressed by a proprietary mobile application that incorporates CGM and HR monitoring coupled with food and activity tracking, and generates glucose predictions for food and activity patterns.

We utilized two blood glucose prediction models (“Continuous Glucose Prediction model”/“CGP model” and “Food Recommendation model”/“FR model”). The former utilizes a machine learning-based algorithm that takes into account the user’s previously recorded blood glucose, heart rate, and food logging information to output the user’s predicted blood glucose values in response to food and activity. The latter recommends to the user foods similar to those the user desires to eat, in order to allow the user to choose foods which cause comparatively lower spikes in glucose. Those models are described in detail below.

### Continuous Glucose Prediction (CGP) algorithm

#### Overview

The machine learning-based algorithm has a base of data that is collected from all users, comprising 46,655 days of data from 1978 users; based on an individual’s entered and captured data, the model is fit to their unique glycemic responses to food and exercise as captured by CGM, HR, and food logging data.

The CGP model has two primary utilities. First, this model allows users to predict the glycemic impact of food 2 h into the future, and without consuming the food item(s) in question (“**CGP**”). Second, this model allows users to continuously estimate blood glucose values throughout the day, if provided information about food logging and heart rate alone (“Virtual CGM” or “**VCGM**”). This aspect of the algorithm allows for continued, personalized recommendations in-between CGM usage periods, thus lessening the user burden and cost associated with physical CGM devices.

The CGP model requires a minimum of 5 days of complete data (12 h of HR and CGM data, in addition to logs of all calorie-containing food and beverage, constitute a day of “complete” data), but continues to fit itself to the individual if the individual continues wearing a CGM and heart rate monitor, and logging food.

The CGP model was an RNN model. The RNN model consisted of a single LSTM layer followed by a dense layer with a sigmoid activation function. The LSTM layer allows the model to capture long-term dependencies in the input sequence. We tried both LSTM and GRU layers with different numbers of nodes and layers. Our evaluation of our model showed that, based on our data, LSTM was the best choice, giving us better RMSE and MAPE.

We furthermore used a meta learning algorithm to optimize the hyperparameters of the RNN model. The meta learning algorithm uses a set of training tasks to learn the optimal hyperparameters for the RNN model. The training tasks consisted of subsets of the dataset. For each training user, the meta learning algorithm generated a set of hyperparameters that optimized the RNN model’s performance on the specific user.

The error in these predictions is 13.4 mg/dl RMSE over the 2 h post-meal period. This error is on par with the best-published results, which do not report this high variance, post-meal period^46^ Notably, the 2-h, post-meal, glucose prediction error remained low at 14.8 mg/dl RMSE, even after participants stopped using their glucose monitors, suggesting that the app was able to learn an individual’s biology sufficiently well so as to predict their glucose response.

#### Data and preprocessing

Our dataset consisted of participant-derived continuous glucose measurements, heart rate readings, physical activity, time stamps, and dietary constituents. This resulted in a time-series database for each variable, offering a rich, multi-modal representation of individual physiological profiles.

Initial preprocessing was conducted to assure the suitability of data for machine learning algorithms. This process comprised the removal of aberrant or incomplete data entries and the standardization of all input features to maintain consistency across the dataset. Additional features were engineered from the raw data to enhance the predictive power of the model. For example, time of day was represented as sin and cos functions to ensure temporal continuity. The preprocessed dataset was bifurcated into a training subset for model learning; and a testing subset for subsequent model performance evaluation.

#### Model architecture

**Fig. 9:**
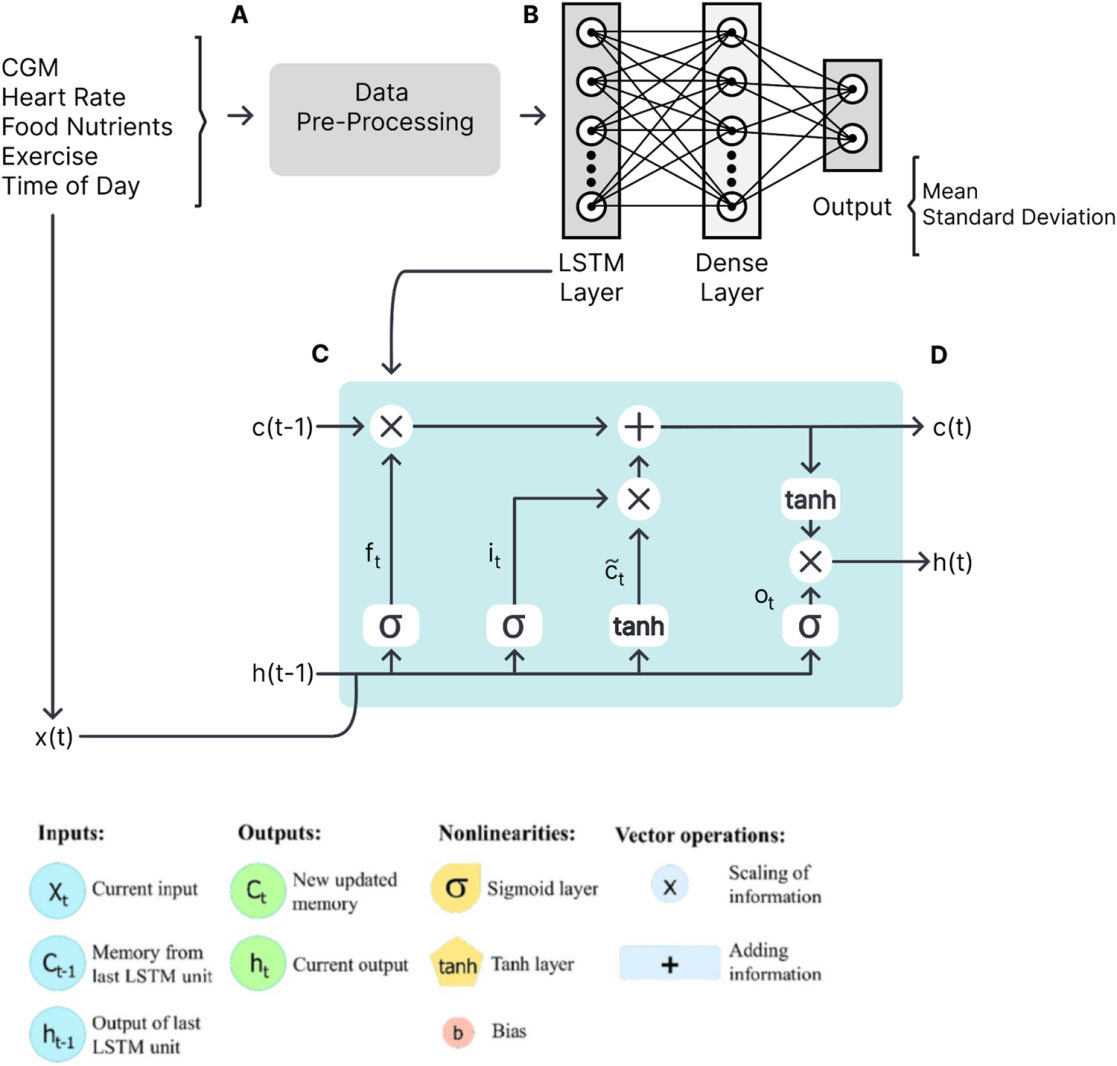
Overview of the Machine Learning Pipeline for Prediction of Blood Glucose Values. This figure illustrates the machine learning pipeline designed for predicting future blood glucose levels based on various inputs, including continuous blood glucose measurements, food nutrients, heart rate, exercise, and time of day. The pipeline consists of several steps, starting with data preprocessing, followed by the utilization of a recurrent neural network (RNN) comprising LSTM (Long Short-Term Memory) and Dense layers. **A** The process begins by collecting and preparing the input data, which encompasses continuous blood glucose readings, food nutrient information, heart rate data, exercise data, and time of day. The collected data then undergoes preprocessing, where it is cleaned, normalized, and organized in a suitable format for the subsequent stages. **B** Next, the preprocessed data is fed into the RNN model, which is composed of LSTM and Dense layers. The LSTM layers are employed to capture temporal dependencies and patterns within the data, enabling the model to understand the sequential nature of blood glucose fluctuations over time. The Dense layers aid in learning complex relationships and extracting relevant features from the input data. **C** The RNN model is trained to predict the blood glucose level for the next time step. Once the initial prediction is made, it is fed back into the model as an input, allowing the model to generate subsequent predictions for future time steps. This feedback loop enables the model to iteratively refine its predictions and adapt to changing conditions. **D** The output of the pipeline is a sequence of predicted blood glucose values, which can be used for various applications, such as monitoring and managing blood glucose levels in individuals with diabetes or supporting personalized dietary and exercise recommendations. Overall, this machine learning pipeline offers a systematic approach for blood glucose prediction, leveraging data preprocessing and a recurrent neural network architecture with LSTM and Dense layers to provide accurate and timely forecasts of blood glucose levels.

The model architecture consists of Long Short-Term Memory (LSTM) layers and Dense layers. The LSTM layers are designed to capture temporal dependencies in the time series data, while the Dense layers provide non-linear transformations and help in the final prediction. The input to the model at each time step includes the following features: continuous glucose values, heart rate, exercise, time of day, and food nutrients. These features are concatenated and fed as input to the model. To train the model, sequences of input data are created from the training set. Each sequence contains a fixed number of consecutive time steps and associated target values (e.g., the next glucose value). The sequences are created by sliding a window over the time series data. The model is trained using the generated sequences from the training set. During training, the model predicts the next step given the current input, and the previous prediction is fed back as an input for the next time step. This feedback loop helps the model learn from its own predictions. The model is trained by minimizing the loss function, log likelihood loss function was used, between the predicted values and the true target values. The ADAM optimizer was used to perform backpropagation and gradient descent algorithms. Hyperparameters, such as the number of LSTM and Dense layers, the size of each layer, learning rate, and batch size, were tuned to optimize the model’s performance. After training, the performance of the model was evaluated using the testing set. Various evaluation metrics, such as root mean squared error, correlation coefficient, and MAPE were computed to assess the accuracy and reliability of the predictions. Based on the evaluation results, further refinements may be made to the model. This could involve adjusting hyperparameters, modifying the architecture, or adding regularization techniques to improve generalization and prevent overfitting. Once the model is trained and evaluated, it can be used to make predictions on new, unseen data. Given a sequence of input features, the model can generate predictions for the next time step(s) of blood glucose values (Fig. 9).

**Fig. 10:**
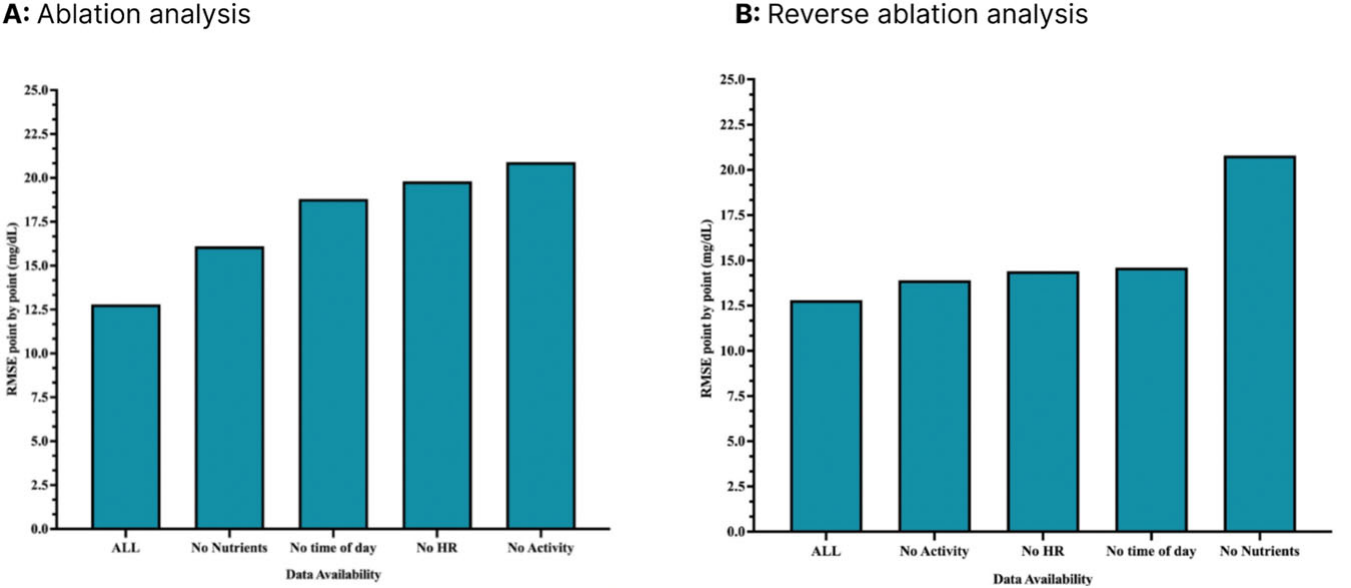
Reliance. **A** Use of ablation analysis to determine feature importance. Starting from the left, the most important modality is removed and the model is retrained to measure the impact of the removed dataset. This analysis demonstrates in order of descending importance the significance of each input was as follows: nutrients, time of day, heart rate, activity. **B** Similar to (**A**) but in each iteration the least important dataset is removed.

We also wished to determine the most significant inputs (among CGM, heart rate, etc.) to the performance of the CGP model. Ablation analysis was conducted (Fig. 10A), removing inputs to the model in sequence to examine the deleterious effects on the model. The difference in the RMSE as a result of ablating nutrient information versus activity information demonstrates that the macronutrients and their quantity consumed are far more important than activity, heart rate, and time of day to the model’s fidelity.

This was followed by reverse ablation analysis (Fig. 10B). While ablation analysis removes inputs to determine which input is most important, reverse ablation analysis adds inputs. The reverse ablation analysis shows that, by adding activity, HR, and time of day information, our RMSE increases from 12.5 to 14. However, upon adding nutrient information, RMSE increases to 21. This demonstrates the dramatic effect that nutrition information has on the fidelity of the model.

Overall, both analyses led us to conclude that, in order of descending importance, the significance of each input was as follows: nutrients, time of day, heart rate, activity.

#### Evaluation of CGP model

##### Data Collected

DID was collected retrospectively from 2217 users over 28 days. Data points included body weight, CGM data, food and activity log data, and HR from an activity tracker. Nutritional breakdown included total caloric intake, macronutrient composition, and fiber intake, which was captured by participants’ self-reported food logs collected in the mobile application. Activity was quantified in minutes/day. Only participants with complete logging and data capture were included in the final analysis (see below). Among those included in the final analysis, adjustments were made to account for potential bias in nutrient intake as a result of differences in logging frequency over time: (1) nutrients were calculated only from “good” logging days, defined as a minimum of two logging events spread throughout waking hours spanning a 16 h range, and a total of ≥1600 calories logged, and (2) specific macronutrients and fiber were presented as grams as well as proportion of total calories (grams converted to calories) such that the changes in the proportion of these nutrients were not biased by residual differences in frequency of logging. Physical activity was adjusted for overall frequency of logging and expressed as adjusted minutes/day. HR data was collected continuously, as was CGM data, both from wearable devices, and did not rely on participant adherence with logging; thus, this data is not subject to logging bias. For CGM, the first day of use is known to be somewhat less accurate, and thus all glycemic measures excluded the first day of use in all participants. Further adjustments were made for days with loss of CGM signal, and this proportion of “lost time” was applied to all measures of event frequency. The average measures such as TIR and GMI did not require this adjustment. HR data capture was remarkably consistent and no gaps in signal were present.

##### Requirements for Inclusion in data analysis

In order to ensure that only individuals who had complete data capture and reasonably consistent data logging were included in the final analysis, the following requirements were designated. Analysis of outcomes included only those individuals who had a sufficient quantity of CGM data capture, consistent food logging, and regular body weight tracking and HR capture. Requirements for CGM data were at least 70% CGM coverage on at least half of the days at the beginning (days 1–5, excluding day 1) and the end (days 15–27) of the 28 day period. Requirements for meal logging were active logging of all meals during the first 7 days, as well as the last 14 days, and HR capture ≥20 h per day. For inclusion in data analysis, users must have logged at least two meals and 1600 kcal/day. Requirements for body weight data tracking were at least one body weight measurement in the first 7 days and in the last 14 days. Because fewer individuals tracked weight at day 28, the analysis of weight change was conducted only in the subset who had the baseline and end of study weight measurements.

##### Statistical analysis

**Fig. 11:**
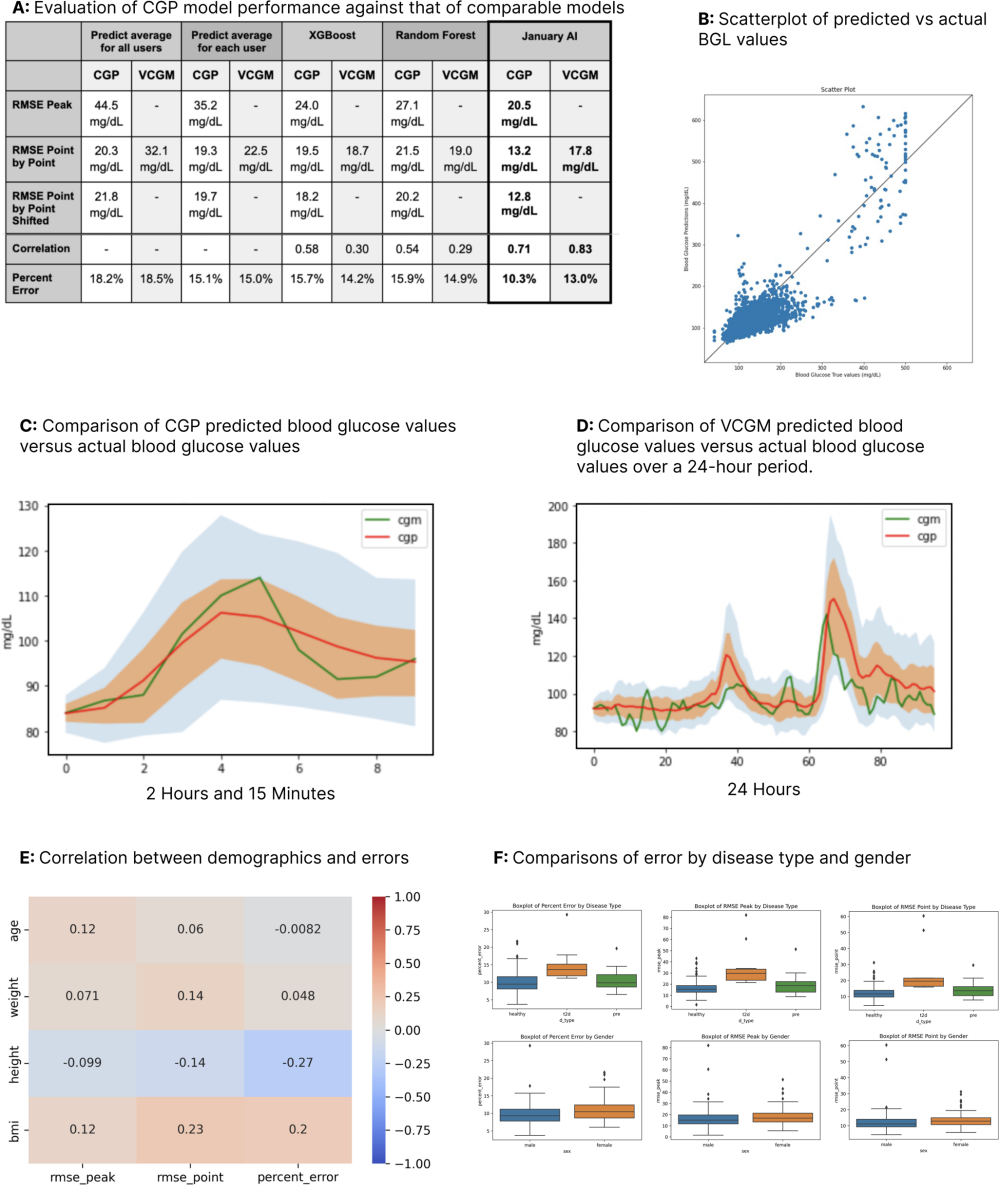
Understanding the performance of the CGP model. **A** Evaluation of CGP model performance against that of comparable models (prediction of the glucose impact of certain foods, “CGP”; and prediction of a glucose curve when given consistent food logging and heart rate information, “VCGM”) to that of several other models. Across a number of dimensions, including RMSE Peak, RMSE point by point, RMSE point by point shifted, correlation, and percent error, we found that the CGP model outperformed each of its competitors. **B** Comparison of actual blood glucose values versus blood glucose values predicted by the CGP algorithm. The correlation coefficient is 0.83, and holds more strongly for non-outlier values (<200 mg/dL). The reasons for this are twofold: first, because BGL fluctuation for these users is generally high; second, because extreme outlier BGL values are rare, and thus appear far less frequently in our training set. **C** Comparison of CGP predicted blood glucose values versus actual blood glucose values. The CGP algorithm operates on a stochastic basis, generating at each 15-min time interval 100 different potential BGL values, along with the corresponding likelihood of each value occurring. The red line reflects CGP predicted BGL; the green line represents BGL values derived from CGM. The orange zone represents the range of BGL values between the 25th and 75th percentiles of the CGP predictions, by likelihood of occurrence; the blue zone represents the 10th and 90th percentiles. As shown, the CGM-derived curve falls within the confidence interval of the predicted curve. **D** Comparison of VCGM predicted blood glucose values versus actual blood glucose values over a 24-h period. The red line reflects CGP predicted BGL; the green line represents BGL values derived from CGM. The orange zone represents the range of BGL values between the 25th and 75th percentiles of the CGP predictions, by likelihood of occurrence; the blue zone represents the 10th and 90th percentiles. As shown, the CGM-derived curve falls within the confidence interval of the predicted curve. **E** Correlation between demographics and errors. We examined whether certain demographic information correlated with higher instances of error. We found that higher weight correlates with higher percent error (*p* < 0.05), and higher age correlates with lower percent error (*p* < 0.05). **F** Comparisons of error by gender and disease type. We found that percent error and RMSE peak/point-by-point were lower for healthy participants than for participants with prediabetes/T2D; and lower for males than for females.

Data was measured using paired-student *t*-tests for beginning vs. end of study for all measures, using Jupyter Notebooks, SciPy, Numpy, Pandas, Matplotlib, Seaborn, pickle, and datetime (the latter two are inbuilt Python packages). Beginning of study was defined as days 2–7 for glucose variables; and days 1–5 for activity and food logging variables. All variables were checked for normality and none needed log transformation for analyses. End of study was defined as days 14–28 for all measures. P < 0.05 was considered statistically significant. The performance of the CGP model was shown to be superior when compared to other models (Fig. 11A), with a high correlation coefficient of 0.833 when comparing actual BGL to predicted BGL (Fig. 11B). A visual depiction of CGP compared to actual curves shows that the actual curve lies within the error bound of the CGP model. Figure 11C shows a comparison of glucose prediction to its corresponding actual CGM curve within a 2-h window, while Fig. 11D shows a comparison of virtual CGM prediction to its corresponding actual CGM curve within a 24-h window. We also examined whether there was a correlation between user demographics and error, in order to determine whether users of certain demographics were more prone to higher error in BGL predictions, and found that higher weight correlates with higher percent error (p < 0.05), and higher age correlates with lower percent error (p < 0.05) (Fig. 11E). Furthermore, we compared error by disease type and gender, in order to determine whether male/female participants, or normoglycemic/prediabetes/T2D participants were more likely to experience higher error in BGL predictions. Our percent error and RMSE peak/point-by-point was lower for healthy users than for users with T2D and with pre-diabetes. Our percent error was also lower for male participants than for female participants, though there are participants whose percent error represents an outlier (Fig. 11F).

#### Food recommendation algorithm

**Fig. 12:**
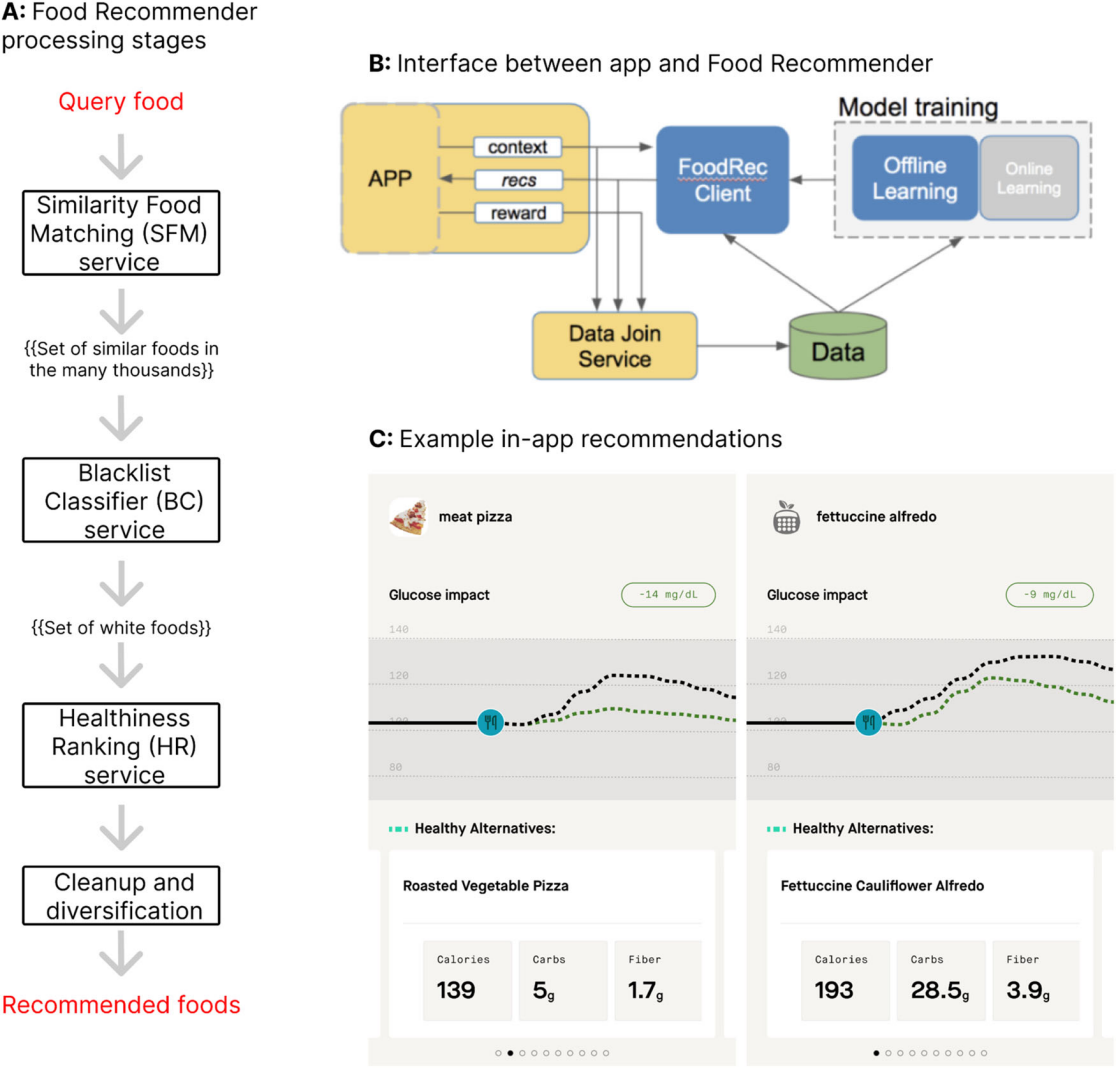
Overview of the Food Recommender model. **A** Food Recommender processing stages. Conceptually, the Food Recommender can be organized as a sequence of 4 processing stages: Input food →(1) Personalized similarity food matching. Finding food items similar to the food item being searched by the user →(2) Blacklist foods removal. Removal of foods that the user has “blacklisted”, i.e. allergies, sensitivities →(3) Healthiness ranking. Ranking of foods according to a “healthiness” scale that takes into account carbohydrate and fiber composition →(4) Cleanup of recommendations list. Removal of substandard recommendations→ Recommendations for user. **B** Interface between app and Food Recommender. **C** Example in-app recommendations. Note the similarity between the food item being looked up, and the food item being recommended to the user.

Food recommendations utilize a separate food recommendation engine underpinned by the CGP model, which predicts the glycemic impact of selected foods based on their macronutrient composition and the individual’s prior responses to macronutrients. The food recommendation engine extrapolates nutritional information and macronutrients from known databases to recommend similar foods that are predicted to be less impactful on a user’s blood glucose, based on the individualized output of the CGP model. The food recommender engine follows four main processing stages, with the user input being a specific food, and the result being similar foods with lower glycemic impacts. The food recommender engine winnows down the food database to find more similar foods, then healthier foods within the set of similar foods (Fig. 12A). Similar, healthier foods are then displayed to the user in-app as “healthy recommendations” (Fig. 12C). The model interfaces with the application via a “FoodRec Client” interface (Fig. 12B).

### Reporting summary

Further information on research design is available in the Nature Research Reporting Summary linked to this article.

### Supplementary information

Supplementary Tables (PDF)

reporting summary

## Data Availability

The data that support the findings of this study are not publicly available due to privacy, commercialization, and/or ethical restrictions. However, data can be made available upon request from the corresponding author.

## Code availability

The underlying code for this study is not publicly available for proprietary reasons.
