# Correction: Digital health application integrating wearable data and behavioral patterns improves metabolic health

**DOI:** 10.1038/s41746-024-00996-y

**Published:** 2024-01-12

**Authors:** Ashkan Dehghani Zahedani, Tracey McLaughlin, Arvind Veluvali, Nima Aghaeepour, Amir Hosseinian, Saransh Agarwal, Jingyi Ruan, Shital Tripathi, Mark Woodward, Noosheen Hashemi, Michael Snyder

**Affiliations:** 1 January AI, Menlo Park, CA USA; 2 Stanford University, Stanford, CA USA

**Keywords:** Lifestyle modification, Patient education

Correction to: *npj Digital Medicine* 10.1038/s41746-023-00956-y, published online 25 November 2023

In this article Ashkan Dehghani Zahedani and Tracey McLaughlin should have been denoted as equally contributing authors. The author order was also incorrect as Tracey McLaughlin should have appeared after Ashkan Dehghani Zahedani but before Arvind Veluvali in the list of authors. Additionally, Tracey McLaughlin should have been listed as co-corresponding the author. The original article has been corrected.

