# Supplementary material for: Digital health application integrating wearable data and behavioral patterns improves metabolic health
Source: NPJ Digit Med. 2023 Nov 25;6:216. doi: 10.1038/s41746-023-00956-y (PMC10673832; doi:10.1038/s41746-023-00956-y)
Supplement: Supplementary file 1 — Supplementary Tables (PDF) [file 41746_2023_956_MOESM1_ESM.pdf]

Supplementary Table 1. **Mean weight loss (lbs) over 12 weeks during Season of Me program**

	Weight Start	Week 2	Week 4	Week 6	Week 8	Week 10	Week 12	12 Week Difference	P-value
**ALL (137)**	176.44	174.22	173.75	173.24	172.08	171.84	172.01	-4.43	1.03E-08
**Healthy (88)**	167.57	166.48	165.90	165.41	165.34	165.22	165.00	-2.57	4.34E-05
**Pre (33)**	188.01	183.99	184.49	184.49	180.08	179.59	181.21	-6.80	3.38E-03
**T2D (16)**	201.32	196.60	194.81	193.08	192.64	192.27	191.58	-9.74	7.33 E-04

Supplementary Table 2. Heart rate changes during Season of Me program

Heart Rate Data: Min/day during which HR was over 110 bpm in the entire cohort (n=1061).

P-values calculated using Student's paired t-test with end-study (28 days) versus baseline data.

	HR >110 Start	HR >110 End	HR >110 Difference	P value
**ALL (1061)**	28.80	32.16	3.36	7.98E-04
**Healthy (764)**	28.33	31.27	2.94	9.41E-03
**Pre (204)**	30.08	36.37	6.29	2.33E-01
**T2D (93)**	29.91	30.31	0.40	0.88

Supplementary Table 3: Nutritional changes during Season of Me program

n	Baseline Cal*	End Cal	Diff Cal	Baseline Carb* (g)	End Carb (g)	Diff Carb (g)	Baseline proportion of carb calories (carb*4/total cal)	End proportion of carb calories (carb*4/total cal)	Baseline Fat (g)	End Fat (g)	Diff Fat (g)	Baseline Sat** (g)	End Sat (g)	Diff Sat (g)	Baseline Protein (g)	End Protein (g)	Diff Protein (g)	Baseline Sugar (g)	End Sugar (g)	Diff Sugar (g)	Baseline Fiber (g)	End Fiber (g)	Diff Fiber (g)	Baseline Proportion of Protein Calories (Protein*4/total cal)	End Proportion of Protein Calories (Protein*4/total cal)	Baseline Proportion of Fat Calories (Fat*9/total cal)	End Proportion of Fat Calories (Fat*9/total cal)	Baseline Proportion of Sat Fat Calories (Sat Fat*9/total cal)	End Proportion of Sat Calories (Sat Fat*9/total cal)
**ALL (542)**	2595	2029	-566	235	179	-56	0.36	0.35	129	102	-27	45	35	-10	126	102	-24	89	61	-28	32	27	-5	0.19	0.20	0.45	0.45	0.16	0.16
**Healthy (392)**	2691	2075	-615	244	184	-59	0.36	0.35	134	105	-29	47	36	-11	128	104	-24	94	63	-30	33	28	-5	0.19	0.20	0.45	0.46	0.16	0.16
**Pre (103)**	2428	1917	-510	206	155	-51	0.34	0.32	122	99	-23	43	33	-11	132	100	-32	77	54	-23	27	23	-4	0.22	0.21	0.45	0.46	0.16	0.15
**T2D (47)**	2366	2084	-281	248	208	-40	0.42	0.40	113	96	-17	39	33	-6	109	97	-14	87	71	-16	36	32	-4	0.18	0.19	0.43	0.41	0.15	0.14

Supplementary Table 4. Standard Deviation of Nutritional changes during Season of Me program

	Baseline Cal*	End Cal	Baseline Carb* (g)	End Carb (g)	Baseline Fat (g)	End Fat (g)	Baseline Sat** (g)	End Sat (g)	Baseline Protein (g)	End Protein (g)	Baseline Sugar (g)	End Sugar (g)	Baseline Fiber (g)	End Fiber (g)
ALL (542)	1041	923	132	103	68	62	32	30	71	55	66	39	27	22
Healthy (392)	1135	985	143	110	73	67	34	33	64	58	75	35	29	25
Pre (103)	844	842	94	97	60	51	28	21	105	53	44	57	18	14
T2D (47)	712	639	120	63	55	45	27	23	37	41	34	21	34	11
